# Internal carotid artery-middle cerebral artery junction small arterial ring: A report of two cases diagnosed by magnetic resonance angiography

**DOI:** 10.1016/j.radcr.2026.08.008

**Published:** 2026-08-31

**Authors:** Akira Uchino, Shunpei Andoh, Nobusuke Tsuzuki

**Affiliations:** a Department of Radiology, Saitama Sekishinkai Hospital, Sayama, Japan; b Department of Neuroendovascular Therapy, Saitama Sekishinkai Hospital, Sayama, Japan; c Department of Neurosurgery, Saitama Sekishinkai Hospital, Sayama, Japan

**Keywords:** Arterial ring, Fenestration, Internal carotid artery, Magnetic resonance angiography, Middle cerebral artery, Volume-rendering image

## Abstract

We herein report 2 cases of internal carotid artery (ICA)-middle cerebral artery (MCA) junction small arterial ring diagnosed by magnetic resonance angiography (MRA). A 73-year-old woman and a 92-year-old man underwent cranial magnetic resonance imaging (MRI) and MRA using a 3-Tesla scanner. MRA was performed using a standard 3-dimensional time-of-flight technique. Cranial MRA showed a small arterial ring at the junction between the ICA and MCA in both cases on maximum-intensity-projection (MIP) images. After the creation of partial volume rendering (VR) images, the variations were clearly identified. The proximal end of the arterial ring was the ICA, and the distal end was the MCA. Using MRA, we diagnosed 2 cases of a small ICA-MCA junctional ring. This variation may not represent ICA or MCA fenestration or MCA duplicate origin. We used the term “ICA-MCA junctional small arterial ring” to describe this variation. Careful observation of MIP images is important for detecting arterial variations on MRA. The creation of partial VR images is useful for the correct diagnosis of arterial variations.

## Introduction

Fenestration occurs when a single artery divides into 2 arterial channels that subsequently fuse. Fenestration of the intradural internal carotid artery (ICA) at the supraclinoid segment, in relation to the origin of the posterior communicating artery (PCoA), is rarely observed [[1], [2], [3], [4]].

There are 2 types of arterial rings in the proximal segment of the middle cerebral artery (MCA): fenestration and duplicate origin. However, these 2 MCA variations are frequently confused and are not differentiated from each other [[5], [6], [7]]. The fusion of 2 different arteries to form an arterial ring represents the duplicate origin of the fused vessel.

We incidentally found 2 cases of small arterial ring at the junction between the ICA and MCA on magnetic resonance angiography (MRA). The proximal end of the arterial ring was the ICA, and the distal end was the MCA. Therefore, this was not a fenestration of the ICA or MCA. There is a possibility of a duplicate origin of the MCA or another type of arterial ring. We used the term “ICA-MCA junction small arterial ring” in this report.

## Case reports

### Case 1

A 73-year-old woman with vertigo and gait disturbance underwent cranial magnetic resonance imaging (MRI) and intracranial MRA using a 3-Tesla scanner (SIGNA Architect; GE Healthcare, Milwaukee, WI). MRA was performed using a standard 3D time-of-flight technique. The imaging parameters were as follows: flip angle, 18°; repetition time, 21.0 seconds; echo time, 3.4 seconds; and slice thickness, 0.8 mm.

**Fig. 1:**
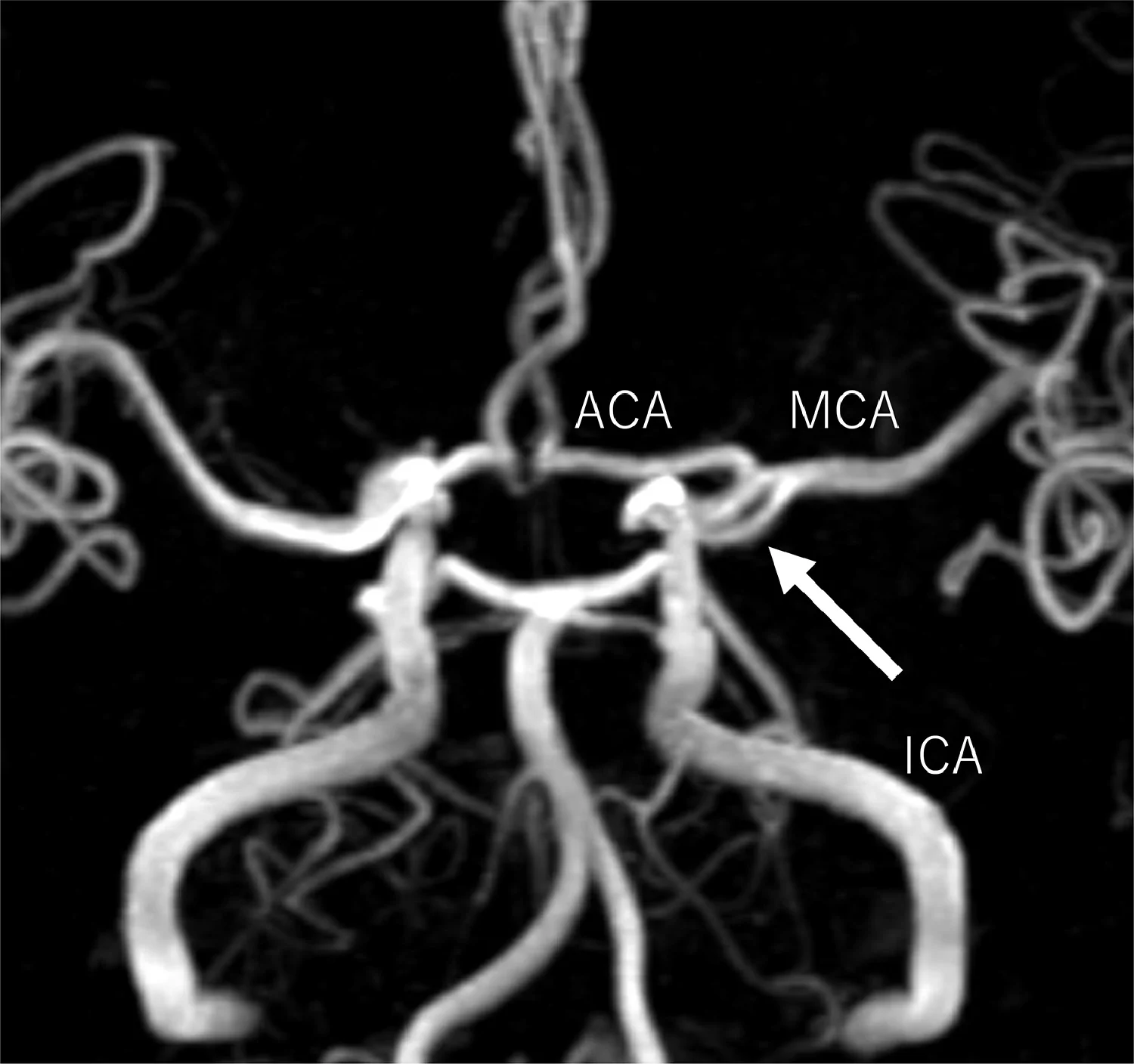
(Case 1) Anteroinferior to posterosuperior projection of maximum-intensity-projection (MIP) image of magnetic resonance angiography (MRA). A small arterial ring is visible at the terminus of the left internal carotid artery (ICA; arrow). ACA, anterior cerebral artery; ICA, internal carotid artery; MCA, middle cerebral artery.

**Fig. 2:**
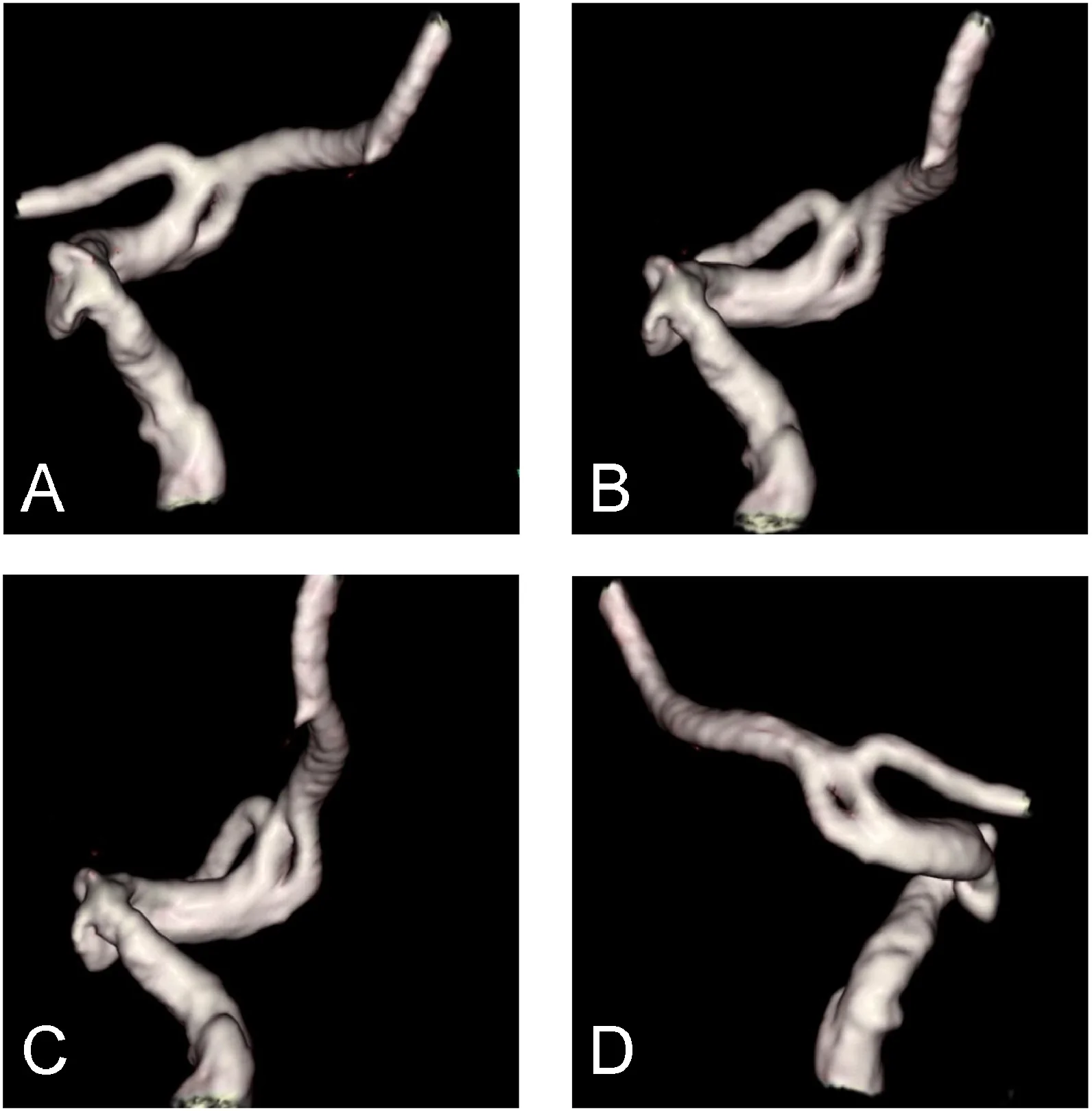
(A-D) (Case 1) Four projections of partial volume-rendering (VR) images of the left carotid system. There is a small arterial ring at the junction between the left ICA and the middle cerebral artery (MCA). Because the proximal end is located at the ICA and the distal end is located at the MCA, this arterial ring is not a fenestration. Although there is a possibility of MCA duplicate origin, this term is not suitable for this variation. Therefore, we propose the term “ICA-MCA junction small arterial ring.”

MRI showed mild white matter ischemic lesions. MRA revealed no significant pathological lesions; however, there was a small arterial ring at the junction between the left ICA and MCA on maximum-intensity-projection (MIP) images (Fig. 1). This finding was clearly identified on partial-volume rendering (VR) images (Fig. 2). Source MRA images were also reviewed by the first author.

No additional radiological examinations, such as computed tomography angiography (CTA) or 3-dimensional rotational angiography (3D-RA), were performed. She was treated conservatively, and her clinical course was good.

### Case 2

A 92-year-old man with disturbed consciousness underwent cranial MRI and MRA using the same machine as in Case 1. MRA was performed using the same technique, and the imaging parameters were the same as those in Case 1.

**Fig. 3:**
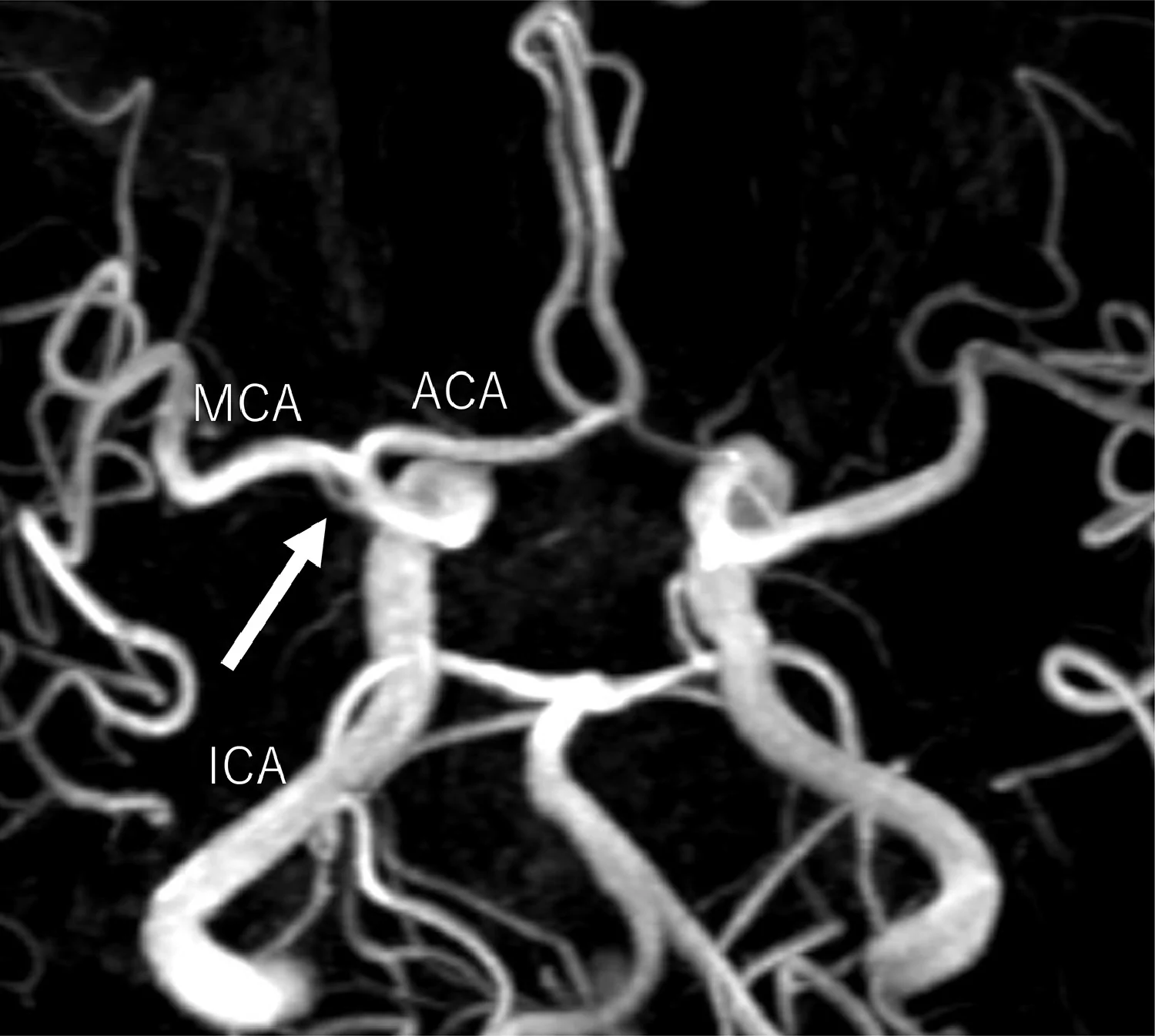
(Case 2) Anteroinferior to posterosuperior projection of MIP image of MRA. A small arterial ring is visible at the terminus of the right ICA (arrow). ACA, anterior cerebral artery; ICA, internal carotid artery; MCA, middle cerebral artery.

**Fig. 4:**
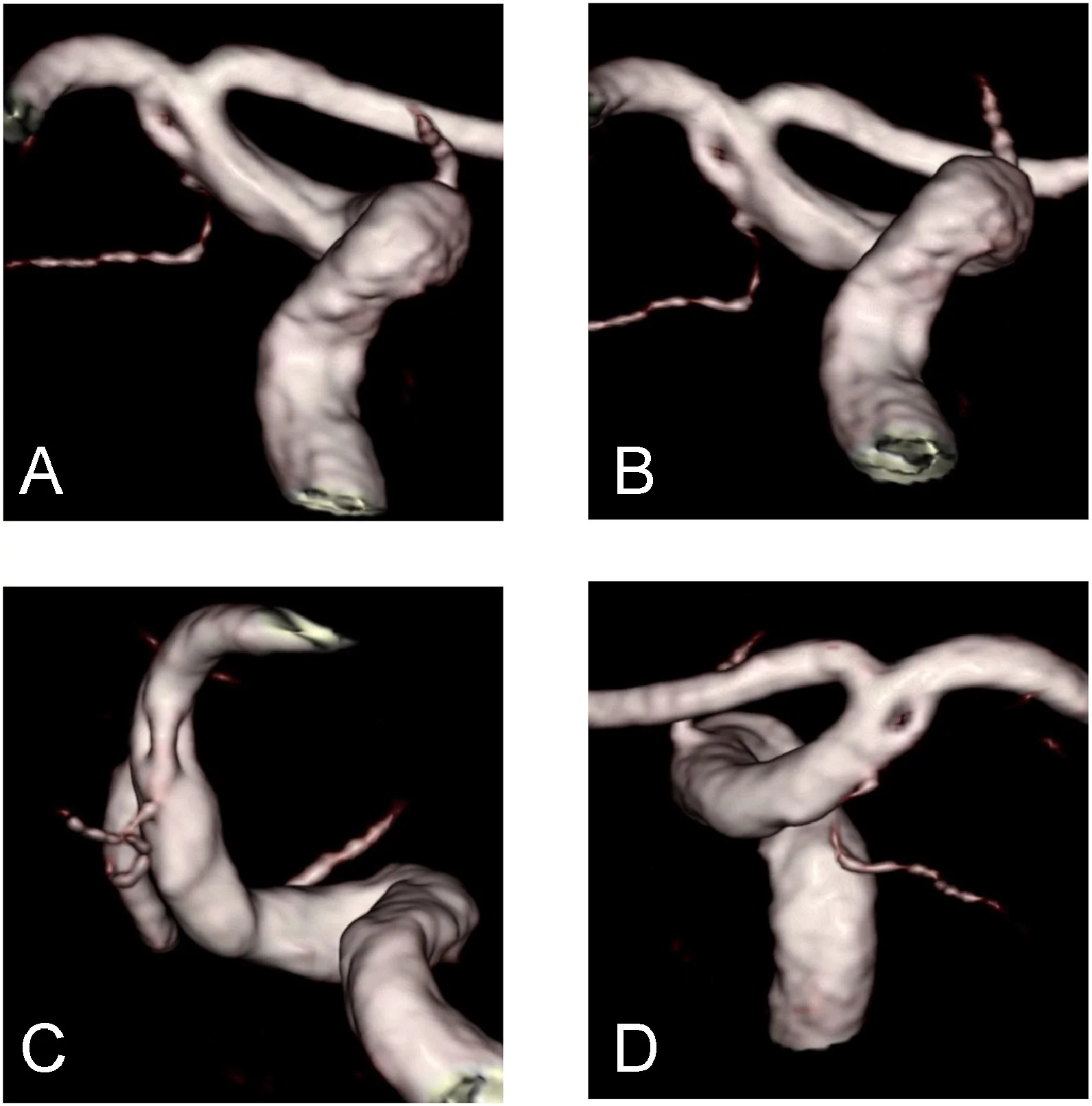
(A-D) (Case 2) Four projections of partial VR images of the right carotid system. A small arterial ring was present at the junction between the right ICA and MCA, similar to that in Case 1.

MRI showed brain atrophy with widespread white matter ischemic lesions. MRA revealed no significant pathological lesions; however, there was a small arterial ring at the junction between the right ICA and MCA on MIP images (Fig. 3). This finding was clearly identified on partial VR images (Fig. 4). Source MRA images were also reviewed by the first author.

No further radiological examinations were performed. He was treated conservatively, and his clinical course was stable.

## Discussion

The primitive arterial network, which is present during early gestation, regresses and fuses to form the adult arterial system. If this regression/fusion fails, various arterial variations may develop. MCA variations, including duplicated origin and fenestration, may result from persistent anastomoses within the primitive vascular network between the anterior cerebral artery and middle cerebral artery. Variations in the site and extent of incomplete fusion may give rise to a wide range of arterial variation [6].

Fenestration is rarely observed in the supraclinoid segment of the ICA. There are several types of fenestrations according to their relationship with the origin of the PCoA [[1], [2], [3], [4]]. An aneurysm is frequently observed at the proximal end of the fenestration. Therefore, if an aneurysm is found at an unusual site in the supraclinoid segment, the presence or absence of a small channel of the fenestration should be checked. In contrast, fenestration is not observed at the terminal segment of the ICA, distal to the origin of the PCoA.

**Fig. 5:**
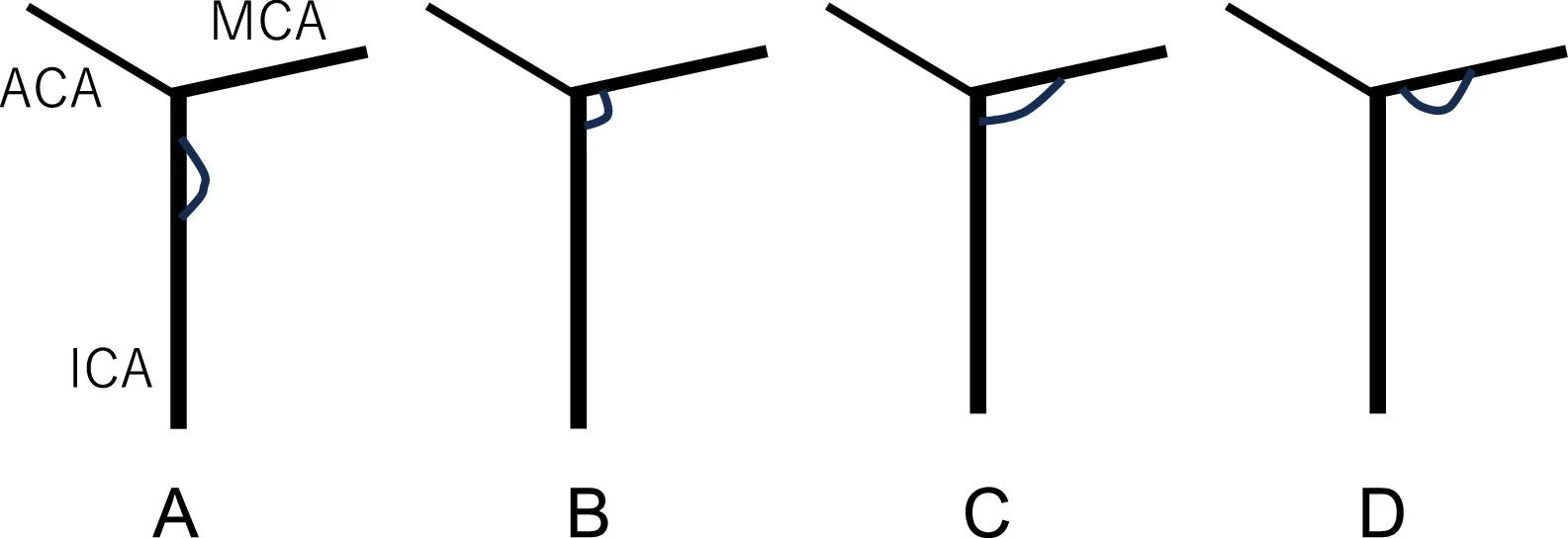
Schematic illustration of the 4 types of arterial rings from distal ICA to proximal MCA. (A) fenestration of the supraclinoid ICA, (B) ICA-MCA junction small arterial ring (present cases), (C) duplicate origin of the MCA, and (D) fenestration of the MCA. ACA, anterior cerebral artery; ICA, internal carotid artery; MCA, middle cerebral artery.

The proximal segment of the MCA contains an arterial ring with fenestration and a duplicate origin. However, duplicate origin is frequently confused with and misdiagnosed as fenestration [[5], [6], [7]]. Using MRA, Zedde and Pascarella [7] recently reported a case of right MCA duplicate origin with a diagnosis of “fenestration.” Triantafyllou et al. [5] recently reported a case of left MCA duplicate origin, forming a small arterial ring at the ICA-MCA junction, similar to our cases. Thus, our 2 cases may be diagnosed as having MCA duplicate origin. However, because the arterial ring is too small and located at the ICA-MCA junction, the term “MCA duplicate origin” may not be suitable for this variation. Therefore, we used the term “ICA-MCA junction small arterial ring” in this report (Fig. 5). However, we agree that this small arterial ring may represent a morphological subtype of duplicate origin of the MCA.

MCA duplicate origin usually forms a relatively small arterial ring. Its prevalence on MRA using a 1.5 T scanner was reported to be 0.11% [6]. No other study has reported the prevalence of MCA duplicate origin. If MRA using a 3T scanner, CTA, or 3D-RA is used for evaluation, a higher prevalence may be reported. Rarely, MCA duplicate origin forms a large arterial ring [8]. MCA duplicate origin is rarely combined with an early bifurcated MCA [9].

MCA fenestration is usually small and is observed at the proximal M1 segment. Its prevalence on MRA using a 1.5 T scanner was reported to be 0.09% [9]. Triantafyllou et al. [5] reported a prevalence of 0.20% for MCA fenestration [6]; however, this is the overall prevalence of duplicate origin (0.11%) and fenestration (0.09%). Therefore, the prevalence of duplicate origin and fenestration is almost equal. The formerly reported prevalence of MCA fenestration, including duplicate origin, was 0.17%-0.43% on digital subtraction angiography and 0.28%-1% on autopsy [10]. Although MRA provides noninvasive anatomical information, 3D-RA remains the gold standard for vascular imaging. In both of our patients, 3D-RA was not performed; therefore, this arterial ring could not be definitively confirmed. Furthermore, if 4-dimensional flow MRI had been performed, hemodynamic assessment might have been possible. Rarely, relatively large fenestrations are found at the mid-M1 segment [11]. MCA fenestration may cause thromboembolic occlusion at the M1 segment because the clot is captured by the fenestrated bifurcation instead of the M1-M2 junction [10,12,13].

The clinical significance of an arterial ring is limited. In both patients, the clinical symptoms, including vertigo, gait disturbance, and impaired consciousness, were unlikely to be related to the arterial rings. Therefore, the arterial rings were considered incidental findings. However, an arterial ring is important in cases of occlusion of the unilateral channel because another patent channel may play a role as collateral circulation. To identify rare arterial variations on MRA, careful observation of MIP images is important, and the creation of partial VR images is useful.

## Conclusions

Using MRA, we diagnosed 2 cases of a small ICA-MCA junctional ring. This vascular variation may not represent ICA fenestration, MCA fenestration, or duplicate origin of the MCA. However, it may represent a morphological subtype of duplicate origin of the MCA. Careful observation of MIP images is important to detect arterial variations on MRA. The creation of partial VR images is useful for the correct diagnosis of arterial variations.

## Patient consent

The patient in Case 1 provided informed consent for the publication of her data and figures. The family of the patient in Case 2 provided informed consent for the publication of his data and figures.
